# Universal Surface Biotinylation: a simple, versatile and cost-effective sample multiplexing method for single-cell RNA-seq analysis

**DOI:** 10.1093/dnares/dsac017

**Published:** 2022-06-02

**Authors:** Michihiko Sugimoto, Yuhki Tada, Shigeyuki Shichino, Saeko Koyamatsu, Noriyuki Tsumaki, Kuniya Abe

**Affiliations:** Technology and Development Team for Mammalian Genome Dynamics, RIKEN BioResource Research Center, Tsukuba City, Ibaraki 305-0074, Japan; Technology and Development Team for Mammalian Genome Dynamics, RIKEN BioResource Research Center, Tsukuba City, Ibaraki 305-0074, Japan; Division of Molecular Regulation of Inflammatory and Immune Diseases, Research Institute of Biomedical Sciences, Tokyo University of Science, Chiba, Japan; Department of Clinical Application, Center for iPS Cell Research and Application, Kyoto University, Sakyo-ku, Kyoto 606-8507, Japan; Department of Tissue Biochemistry, Graduate School of Medicine and Frontier Biosciences, Osaka University, 2-2 Yamadaoka, Suita, Osaka 565-0871, Japan; Department of Clinical Application, Center for iPS Cell Research and Application, Kyoto University, Sakyo-ku, Kyoto 606-8507, Japan; Department of Tissue Biochemistry, Graduate School of Medicine and Frontier Biosciences, Osaka University, 2-2 Yamadaoka, Suita, Osaka 565-0871, Japan; Technology and Development Team for Mammalian Genome Dynamics, RIKEN BioResource Research Center, Tsukuba City, Ibaraki 305-0074, Japan; Life Innovation Program, University of Tsukuba, Tsukuba City, Ibaraki 305-8577, Japan

**Keywords:** single-cell RNA-seq, multiplexing, biotinylation

## Abstract

Recent advances in single-cell analysis technology have made it possible to analyse tens of thousands of cells at a time. In addition, sample multiplexing techniques, which allow the analysis of several types of samples in a single run, are very useful for reducing experimental costs and improving experimental accuracy. However, a problem with this technique is that antigens and antibodies for universal labelling of various cell types may not be fully available. To overcome this issue, we developed a universal labelling technique, Universal Surface Biotinylation (USB), which does not depend on specific cell surface proteins. By introducing biotin into the amine group of any cell surface protein, we have obtained good labelling results in all the cell types we have tested. Combining with DNA-tagged streptavidin, it is possible to label each cell sample with specific DNA ‘hashtag’. Compared with the conventional cell hashing method, the USB procedure seemed to have no discernible adverse effect on the acquisition of the transcriptome in each cell, according to the model experiments using differentiating mouse embryonic stem cells. This method can be theoretically used for any type of cells, including cells to which the conventional cell hashing method has not been applied successfully.

## 1. Introduction

Global gene expression analysis is an indispensable technique for analysing the characteristics of cells and tissues. Comparisons of gene expression profiles within various cells and tissues can reveal the diversity of cell types present and the characteristics and proportions of different cells within different tissues. In conventional bulk analysis, a large number of cells are mixed together, and mRNA is extracted from the mixed samples for expression analysis, which reveals the average gene expression in many of these cells. However, biological tissues are complex and consist of a wide variety of cell populations. Thus, following the bulk analysis of whole tissues, it is difficult to accurately interpret cellular states, and to determine the genes expressed and their functions. Even cultured cell lines that are thought to be homogeneous can be heterogeneous populations, and thus it is impossible for bulk RNA-seq analysis to accurately capture changes in gene expression that vary with the cell cycle or other modalities.

However, single-cell RNA sequence (scRNA-seq) analysis is an extremely useful genomic technique to overcome these problems and represents an essential genomic tool for dissection of complex biological phenomena.^1^ scRNA-seq involves capturing single cells and creating an RNA-seq library from each cell for transcriptome analysis. Nonetheless, using a large number of cells as a sample was until recently both labour intensive and costly, and scRNA-seq has not been an easily applied technology. However, technologies such as droplet scRNA-seq using microfluidics/microdroplet reaction systems or microwells have now been developed, and equipment for these technologies is commercially available (e.g. 10× Chromium,^2^ BD Rhapsody system^3^). These advances have enabled the preparation of scRNA-seq libraries from 10,000 cells or more, and the cost of analysis per cell has been greatly reduced.^4^ However, although the cost per cell can be reduced, the analysis cost as a whole will remain high if multiple samples are analysed or a time series analysis at multiple time points is conducted. In addition, when sample preparation and scRNA-seq experiments are performed at different times, errors (i.e. batch effects) can occur in the analysis of each experimental batch.^5^ Therefore, if multiple samples can be grouped together and processed simultaneously in a single library preparation, not only can the analysis cost be greatly reduced, but batch effects can be reduced and more precise scRNA-seq analysis can be performed. This technique for grouping multiple samples together and analysing them in a single experimental operation is called sample multiplexing. In the multiplexing method, multiple cell populations are prelabelled with barcode DNA tags (=hashtag) that have unique sequences for each sample, and then mixed for single-cell analysis. The obtained RNA-seq data can be used to trace the origin of each cell based on the hashtag DNA sequence. scRNA-seq analysis using droplet sequencing or microwells has the potential to trap multiple cells in a single compartment and such doublet or multiplet errors make data interpretation difficult. However, if different samples can be distinguished by the DNA hashtags, the multiplet error can be reduced, and there is a precedent for multiplexing using this principle. Methods using a cell-labelling antibody conjugated with a hashtag DNA that recognizes a specific cell surface protein has been reported.^6^ However, a problem with this cell hashing method is that it assumes that the cell surface protein in question is expressed by all the cells in the test samples. The Cell-Hashtag antibody is a cocktail of antibodies intended to universally label cells of adult tissue origin (e.g. TotalSeq anti-mouse Hashtag Antibody, BioLegend). In the case of Hashtag, antibodies against CD45 and MHC class I are used (the former is for detecting cells of the immune system, while the latter is for differentiated cells, in general). However, pluripotent stem cells, for example, which are often used as a model of embryonic development, do not express CD45 or MHC class I, and the abovementioned method is not applicable.^7^^,^^8^ We have also found that the method is not applicable to some nonembryonic cells, such as cartilage cells.

Therefore, in this study, we developed the Universal Surface Biotinylation (USB) method as a new cell labelling technique for multiplex scRNA-seq analysis that is not dependent on specific cell surface antigens and demonstrated that it is applicable to the analysis of differentiating embryonic stem (ES) cells. This method combines the labelling of cell surface proteins with S-NHS-biotin and streptavidin with a DNA-hashtag, and the USB method is a universal labelling method that can be theoretically applied to any type of cells and represents a simple, highly efficient and cost-effective method for multiplexing scRNA-seq.

## 2. Materials and methods

### 2.1. Cell culture

R1 ES cells were generously provided by Dr. Sado (Kindai University, Japan). EB3 ES cells (AES0139)^9^^,^^10^ were provided by the Cell Bank of the RIKEN BioResource Research Center. Undifferentiated ES cells were maintained according to methods described previously,^11^ with minor modifications. Briefly, ES cells were cultured in Glasgow Minimal Essential Medium (GMEM; Merck, G5154) supplemented with 14% KnockOut Serum Replacement (Thermo Fisher Scientific, 10828028), 1% foetal bovine serum (FBS), 1,000 U/ml leukaemia inhibitory factor, 0.11 mg/ml sodium pyruvate (Nacalai Tesque, 29806-12), 0.1 mM 2-mercaptoethanol (Thermo Fisher Scientific, 21985023), 1× nonessential amino acids (Thermo Fisher Scientific, 11140076) and 1× GlutaMAX supplement (Thermo Fisher Scientific, 35050061) on mitomycin C-treated mouse embryonic fibroblast (MEF) feeder cells. For passaging, 0.25% trypsin (Fuji Film, 201-16945) was used.

### 2.2. Induction of ES cell differentiation

After removing feeder cells, 1 × 10^4^ ES cells were seeded in low cell-binding U-bottom 96-well plates (Nunclone Sphera 96-Well U-Shaped-Bottom Microplate; Thermo Fisher Scientific, 174925) in 100 µl of the differentiation medium (GMEM supplemented with 15% FBS, 0.11 mg/ml sodium pyruvate, 0.1 mM 2-mercaptoethanol, 1× nonessential amino acids and 1× GlutaMAX supplement) and cultured for 24 h. Spheroids made in the plate were transferred to low cell-binding 90 mm dishes (Nunclon Sphera 90 mm dish, 174945) and cultured for a further 4 days to form embryoid bodies (EBs). EBs were placed onto gelatin-coated culture dishes and cultured for 7 days to induce further differentiation.

### 2.3. Preparation of single cells for scRNA-seq analysis

ES cells were dissociated into single cells and cultured for 1 h on gelatin-coated culture dishes to remove feeder cells. Floating cells were collected as undifferentiated ES cells. After washing three times with PBS, differentiated cells were treated with 0.25% trypsin and dissociated into single cells. Dead cells contained in the cell suspension were removed by Percoll centrifugation. Briefly, cells were suspended in 25% Percoll PLUS (Cytiva, 17544501) in RPMI 1640 (Fuji Film, 183-02165) supplemented with 5% FBS and 10 mM HEPES (Nacalai Tesque, 17557-94) and overlaid on 65% Percoll PLUS. After centrifugation at 1,000 *g* for 20 min, the interphase between the 25% and 65% Percoll solutions was collected and washed with differentiation medium. The cell suspension was filtered with a 40 µm cell strainer, and the cell viability was measured as the ratio of trypan blue-negative cells.

### 2.4. USB labelling

S-NHS-biotin stock solution was prepared by dissolving EZ-Link sulfo-NHS-biotin (Thermo Fisher Scientific, 21217) in PBS at a 10 mg/ml concentration and aliquots were stored at −80°C until use. Cells (0.5–2 × 10^6^) were washed once with ice-cold PBS supplemented with 1% FBS and suspended in 500 µl of ice-cold S-NHS-biotin working solution in PBS with 1% FBS (in this study, the concentration was 10 µg/ml, but the range of the S-NHS-biotin concentration should be 10–50 µg/ml, depending on the cell types studied). After incubation for 10 min on ice, 3 ml of ice-cold PBS with 3% FBS was added to the reaction, and cells were centrifuged for 5 min at 300 *g* at 4°C. Cells were washed two times further with Cell Staining Buffer (BioLegend, 420201) and kept on ice until the next step of hashtag oligo DNA binding.

### 2.5. Ab labelling with anti-CDH1 antibody

Cells (0.5–2 × 10^6^) were washed once with ice-cold Cell Staining Buffer and suspended in 50 µl of ice-cold blocking solution [0.05 µg/ml of TruStain FcX PLUS antibody (BioLegend, 156603) in Cell Staining Buffer], and incubated for 10 min on ice. A 50 µl aliquot of ice-cold primary antibody solution [0.05 µg/ml of biotinylated CDC324 (E-cadherin) rat monoclonal antibody (Thermo Fisher Scientific, 13-3249-82) in Cell Staining Buffer] was mixed with the cell suspension, and cells were incubated for 30 min on ice followed by washing three times with ice-cold Cell Staining Buffer and kept on ice until hashtag DNA binding. Biotinylated rat IgG1 isotype control (Thermo Fisher Scientific, 13-4301-82) was used as an isotype control.

### 2.6. Hashtag DNA binding

In this study, barcode DNA for cell hashing (sample multiplexing) is called ‘hashtag’. The hashtag DNA contains barcode sequence of 15 nucleotides (Supplementary Table S1). TotalSeq PE streptavidin or TotalSeq anti-biotin antibody conjugated with a hashtag DNA (BioLegend, see Supplementary Table S1) was used to transfer hashtag DNA to biotin-labelled cells as follows. Cells were suspended in 100 µl of ice-cold 0.6 µg/ml TotalSeq PE streptavidin or 2 µg/ml TotalSeq anti-biotin antibody, diluted with Cell Staining Buffer and incubated for 30 min on ice, followed by 3× washes. The cell suspension was filtered with a 40 µm cell strainer, and equal numbers of cells from each sample were combined into a single tube, and used for cell capture and cDNA synthesis with the BD Rhapsody apparatus.

### 2.7. Flow cytometry

Single-cell suspensions of undifferentiated and differentiated ES cells were prepared as described above. Normal F344 rat lung was minced into 0.5 mm^2^ with a razor blade and digested with Liberase solution [RPMI-1640 (Nacalai Tesque) supplemented with 10% FBS, 10 mM HEPES pH7.2–7.4, 0.25 mg/ml Liberase TM (Roche), and 2000 U/ml DNase I (Merck)] at 37°C for 60 min to prepare single-cell suspension. Dead cells were removed with Percoll. Growth plate cartilage from proximal tibia were collected from 3-week-old mice and minced into 1–2 mm pieces, and then dissociated into single cells with the Liberase solution for 120–210 min with sustained shaking. Reagents for cell staining were Biotinylated CDC324 (E-cadherin) rat monoclonal antibody (Thermo Fisher Scientific, 13-3249-82), TotalSeq PE streptavidin (BioLegend, see Supplementary Table S1), PerCP-Cy5.5 anti-Rat CD45 (Bio-Rad, clone OX-1), PE anti-Rat CD31 (Bio-Rad, clone TLD-3A12), Streptavidin-APC (BioLegend, 405207), APC Rat anti-Mouse CD45 (BD Bioscience, 559864) and PE anti-Mouse H-2 Antibody (BioLegend, Inc., 125505). Stained cells were analysed by flow cytometry using the EC800 Cell Analyzer (Sony), CytoFLEX flow cytometer (Beckman Coulter) or FACS Aria II flow cytometer (BD Biosciences).

All animal experiments were reviewed and approved by the institutional animal committee of Kyoto University and by the Animal Experiment Committee of Tokyo University of Science (approval number: S17034, S18029, S19024 and S20019).

### 2.8. Immunofluorescence

Cells cultured on gelatin-coated glass-bottom dishes were fixed with 4% paraformaldehyde in PBS at 4°C overnight, followed by 3× washes in wash buffer (0.1% Triton X-100 in PBS). Cells were incubated in blocking buffer (PBS with 1% BSA and 0.1% Triton X-100), and then incubated with primary antibodies diluted in blocking buffer at 4°C overnight. After washing with wash buffer, cells were incubated with secondary antibodies diluted with blocking buffer at room temperature for 1 h, and mounted with 40% glycerol in PBS containing 2% 1,4-Diazabicyclo [2.2.2] octane (DABCO) (Sigma, D2522). Images were captured using a LEICA AF6500 fluorescence imaging system (Leica). Antibodies used in this research were as follows: primary antibodies—biotinylated CDC324 (E-cadherin: CDH1) rat monoclonal antibody (Thermo Fisher Scientific, 13-3249-82) (1:200) and anti-OCT4 rabbit polyclonal antibody (Abcam, ab19857) (1:400); and secondary antibodies—Alexa Fluor 488 anti-rat donkey IgG (Thermo Fisher Scientific, A21208) (1:500) and Alexa Fluor 555 anti-rabbit donkey IgG (Thermo Fisher Scientific, A31572) (1:500).

### 2.9. Cell viability test

Undifferentiated ES cells were treated with 100 µg/ml of S-NHS-biotin (10 times higher concentration than that used for multiplex scRNA-seq) and TotalSeq PE streptavidin, and 3 × 10^3^ cells were seeded onto a gelatin-coated 3.5 cm culture dish. In parallel, the same number of untreated cells were seeded. After culture for 5 days, cells were stained with haematoxylin and the colony formation efficiencies of untreated control and treated samples were examined.

### 2.10. scRNA-seq

To prepare the scRNA-seq library, a BD Rhapsody Express Single-Cell Analysis system (BD Biosciences) and parts of a Targeted mRNA and AbSeq Amplification Kit (BD Biosciences, 633771) were used. Cells (1.6 × 10^4^) labelled with hashtag DNA-streptavidin or antibody were trapped in a microwell cartridge, and cell capture beads capturing single-cell-derived mRNA and hashtag oligo DNA were collected. Using ∼25% of the beads (∼4,000 cells equivalent), cDNA synthesis was conducted according to the instructions for the BD Rhapsody Express. cDNA and hashtag DNA were amplified using the TAS-seq protocol, as described in ref.^12^ Briefly, cell capture beads binding cDNA and hashtag DNA were added to poly-C tail by treating with 0.75 U/µl of terminal deoxynucleotidyl transferase (TdT; Enzymatics, P7070L), 1 mM dCTP (GE Healthcare, 28406512) and 0.05 mM ddCTP (GE Healthcare, 27206101) in 1× TdT buffer (Thermo Fisher Scientific, 16314015) for 30 min at 37°C with mixing at 1,200 rpm. To synthesize second-strand cDNA sequences, the poly-C-tailed cDNA beads were treated with 1× KAPA HiFi HS ReadyMix (Roche, 7958935001) with 5′-BDWTAv2-9G primer using the programme of (98°C for 20 s, 47°C for 1 min, 72°C for 2 min) × 16 cycles. And then, 1× KAPA HiFi HS ReadyMix with three types of primers (5′-BDWTAv2, Universal Oligo-long, and TotalSeq-ADT-oligo 1) was added to the reaction, and the whole transcriptome was amplified using the programme of (98°C for 20 s, 63°C for 20 s, 72°C for 5 min) × 7 cycles. Amplified cDNA and hashtag DNA were separated by size selection using AMPure XP beads (Beckman Coulter, A63881). cDNA was further amplified by treatment with 1× KAPA HiFi HS ReadyMix with two primers (5′-BDWTAv2 and Universal Oligo-long) using the programme of (98°C for 20 s, 65°C for 20 s, 72°C for 5 min) × 5 cycles, and hashtag DNA with two primers (TotalSeq-ADT 2 and Universal Oligo-long) (98°C for 20 s, 65°C for 20 s, 72°C for 5 min) × 12 cycles. Primers used for TAS-seq amplification are listed in Supplementary Table S1. Amplified sequences were purified using AMPure XP beads, and the size distribution and yield were checked using Bioanalyzer (Agilent) with a High Sensitivity DNA Kit (Agilent, 5067-4626). Acquisition of sequencing data was done by ImmunoGeneTeqs, Inc., using a NovaSeq 6000 S4 flow cell (Illumina).

### 2.11. Data analysis

The de-multiplexed and DBEC-treated sequencing data were imported into R (4.0.2), and cells that were determined to be ‘doublet’ or ‘not detected’ from the hashtag count analysis, and cells with a small number of reads were deleted. Genes with a small number of reads or genes that were not found in a sufficient number of cells were deleted. Cells with more than 10% mitochondrial gene counts were also filtered out (these cells were considered as dead cells in this study). Starting from ∼4,000 input cells, number of cells with cell ID barcodes^3^ were found to be 2,864, of which 353 were classified as dead cells, 15 were doublets and 150 were cells with a small number of reads or genes. We found that 342 cells had scarce hashtag counts likely due to non-specific binding of anti-CDH1 antibody or hashtag-Streptavidin, or very low-level expression of the target molecule (see Supplementary Fig. S1). Thus these 342 cells cannot be used reliably for multiplexing. After deducting these non-informative cells, the remaining 2,004 cells were used for the subsequent informatics analysis using the Seurat package (4.0.5), following the workflow in the tutorial, with modifications to parameters.^13^^,^^14^ The count matrix was then used to perform the default global scaling normalization method, LogNormalize. FindVariableFeatures was used to calculate the top 2,000 highly variable genes using the selection method, vst. After scaling the data, Principal Component Analysis (PCA) was performed. Based on the Euclidean distance in space occupied by the first 30 principal components identified, a K-nearest neighbour graph was constructed, and the Louvain algorithm was applied to identify clusters. For the uniform manifold approximation and projection (UMAP) analysis of six samples, 2,000 highly variable genes, 30 principal components (PC) and a resolution of 0.5 were used to obtain 10 clusters. For analysis of four samples, i.e. USB-treated undifferentiated R1, USB-treated differentiated R1, Ab-treated undifferentiated R1 and Ab-treated differentiated R1, 3,000 variable genes, 50 PC and resolution 0.5 were used to obtain 8 clusters. Marker genes for each cluster were identified using FindAllMarkers (min.pct = 0.25, logfc.threshold = 0.25, set only.pos = TRUE). *Cdh1* expression was plotted in FeaturePlot.

Differential gene expression testing was performed using the non-parametric Wilcoxon rank sum test and MAST^15^ of Seurat.^13^^,^^14^

### 2.12. Data availability

All raw FASTQ sequencing files have been deposited to DNA Data Bank of Japan (DDBJ; https://www.ddbj.nig.ac.jp/index.html 7 June 2022, date last accessed) under the accession numbers DRR333192-DRR333193.

## 3. Results

### 3.1. Principle of cell labelling by the USB method

**Figure 1:**
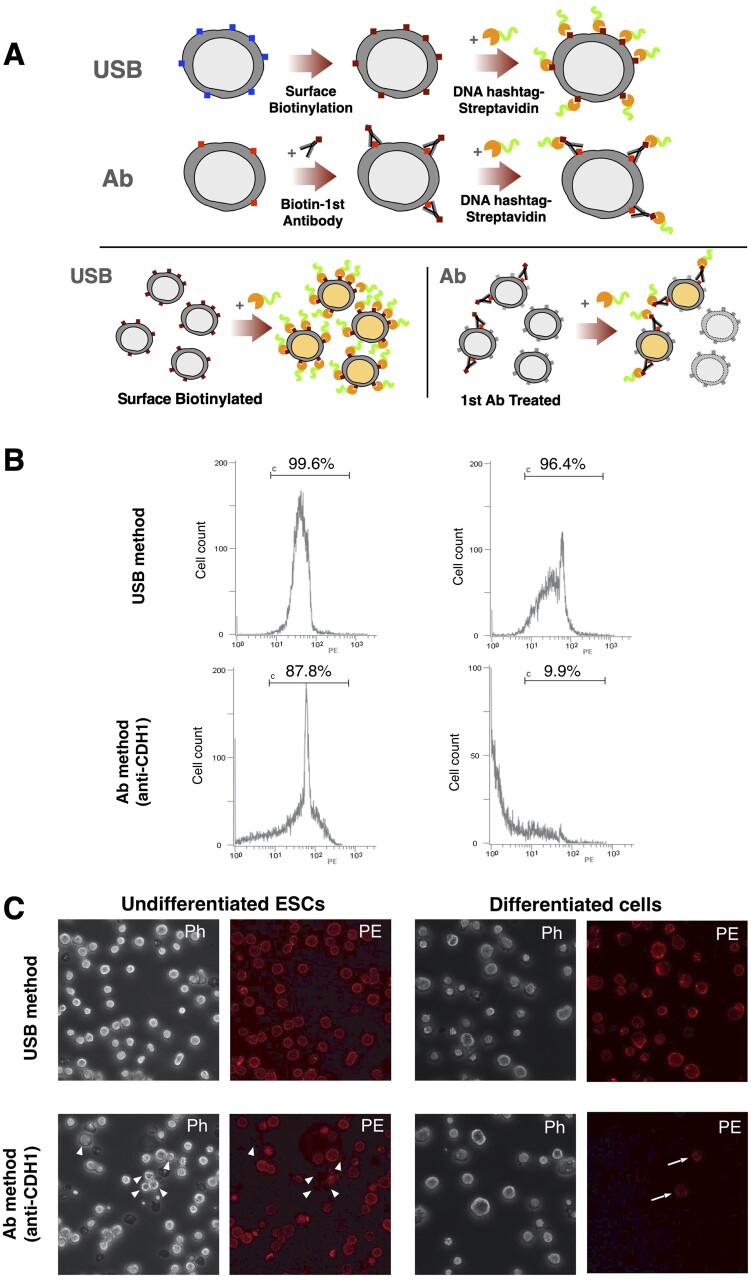
Principle of USB method and comparison of USB and Ab labelling methods. (A) Principle of the USB and Ab methods (top). In the USB method, cell surface proteins (represented by small rectangles on the cell) are universally biotinylated with S-NHS-biotin and treated with hashtag DNA-tagged streptavidin. In the Ab method, cells are treated with biotin-conjugated antibody against specific cell surface protein (small rectangles on the cell) followed by the hashtag DNA-tagged streptavidin. If the specific protein is not expressed on some cells, those cells are not labelled by the Ab method (bottom right), whereas the USB method can label all the cells (bottom left). (B) Efficiency of cell labelling examined by flow cytometry. The USB labelling is highly efficient, with 99.6% of undifferentiated (top left) and 96.4% of differentiated cells (top right) being PE positive. In contrast, using the Ab method, 87.8% of undifferentiated cells (bottom left) and 9.9% of differentiated cells (bottom right) were PE positive. (C) Cell labelling by the USB method (top panel) and the Ab method (bottom panel) confirmed by fluorescence microscopy. In this case, the Ab method uses an antibody against mouse CDH1. CDH1 is known to be expressed in the undifferentiated ES cells, while the expression is limited to some cells in differentiated states. (Left) Undifferentiated ES cells; (right) differentiated cells (12 days after induction of differentiation). ph, phase-contrast images; PE, fluorescence images of TotalSeq-PE-streptavidin; arrowhead, PE negative cells in left panel; arrow, PE positive cells in right panel.

We have developed a new cell labelling technique for multiplex scRNA-seq using S-NHS-biotin, designated as the USB method (Fig. 1A). This method is a universal method that does not require specific surface antigens because it targets amine groups present in almost all proteins, whereas the conventional method (designated here as Ab method) targets specific proteins on the cell surface.^6^ In the Ab method, cells cannot be labelled if the target surface protein is not expressed, whereas the USB method allows labelling, as long as the surface protein is present (Fig. 1A).

In this study, as a proof-of-concept experiment, we compared two methods for analysis of undifferentiated mouse ES cells and their differentiated derivatives. In the undifferentiated state, almost all mouse ES cells express the adhesion molecule E-cadherin (CDH1),^16^ but as they differentiate, the number of cells that do not express CDH1 increases (Supplementary Fig. S2A and B). Therefore, the Ab assay using anti-CDH1 antibody labels most cells in the undifferentiated state, but not all the differentiated cells. In contrast, as the USB method does not depend on CDH1 expression, it is predicted to be able to label cells in both undifferentiated and differentiated states.

### 3.2. Cell labelling by USB

Single cells were prepared from undifferentiated mouse ES cells (R1 line) and cells differentiated from R1 ES cells through EB formation, and used for the experiments described. The cell samples were divided into two; one half was treated using the Ab method, i.e. biotin-conjugated, anti-CDH1 antibody, and the other half was treated using the USB method to add biotin to cell surface proteins. Rest of the procedures were the same for the two methods and all the procedures were done on ice with pre-chilled reagents aiming to avoid changes in transcriptome during the experimental procedure. Viabilities of the USB-treated and Ab-treated cells did not change significantly before and after the treatments (Supplementary Fig. S2C), suggesting that both methods do not induce noticeable cell death. To examine long-term effects, if any, on cell viability, cells treated with S-NHS-biotin were seeded into dishes, and their colony-forming ability was measured. As shown in Supplementary Fig. S2D–F, there was no significant differences in colony numbers between the treated and untreated cells.

Since the hashtag DNA-tagged streptavidin used is conjugated with the fluorescent dye phycoerythrin (PE), flow cytometry analysis was performed to examine the labelling efficiency (Fig. 1B). Using Ab labelling, the percentage of PE-positive cells in undifferentiated ES cells was 87.8%, while that in differentiated ES cells was ∼10%. Immunostaining with anti-CDH1 antibody revealed that almost all cells in the undifferentiated ES cell samples were positive for CDH1 (Fig. 1C), but CDH1 expression was only detected in some of the differentiated cells, which was consistent with the flow cytometry results. In contrast, using the USB method, the percentage of PE-positive cells in the undifferentiated ES cell and differentiated cell samples was 99.6% and 96.4%, respectively (Fig. 1B and C). Fluorescence microscopy also demonstrated that almost all cells were fluorescently labelled (Fig. 1C, upper panel). These results indicate that the USB method is capable of efficiently labelling most cells in both undifferentiated and differentiated states.

### 3.3. Sample multiplexing and scRNA-seq analysis using the USB method

As described, the newly developed USB method can universally attach hashtag DNA to most cells via biotin–streptavidin binding, and the experimental manipulation does not impair the viability of cells. Next, we examined whether this technique could be used for multiplexing in scRNA-seq analysis, and whether the expression profiles of cells would be affected by the experimental manipulations. R1 ES cells in undifferentiated and differentiated states were processed by either the USB or Ab method, and undifferentiated and differentiated cells of another ES cell line, EB3, were labelled using the USB method. These six samples were mixed in equal amounts, and about 16,000 single cells were captured using the BD Rhapsody system. A portion of these cells (∼4,000 cells) was used for subsequent cDNA synthesis and amplification for whole-transcriptome analysis and amplification of hashtag DNA according to the TAS-seq protocol.^12^ The size distribution and yield of the amplified cDNA and hashtag oligo DNA were estimated, and appropriate amplification was confirmed (Supplementary Fig. S2G and H).

**Figure 2:**
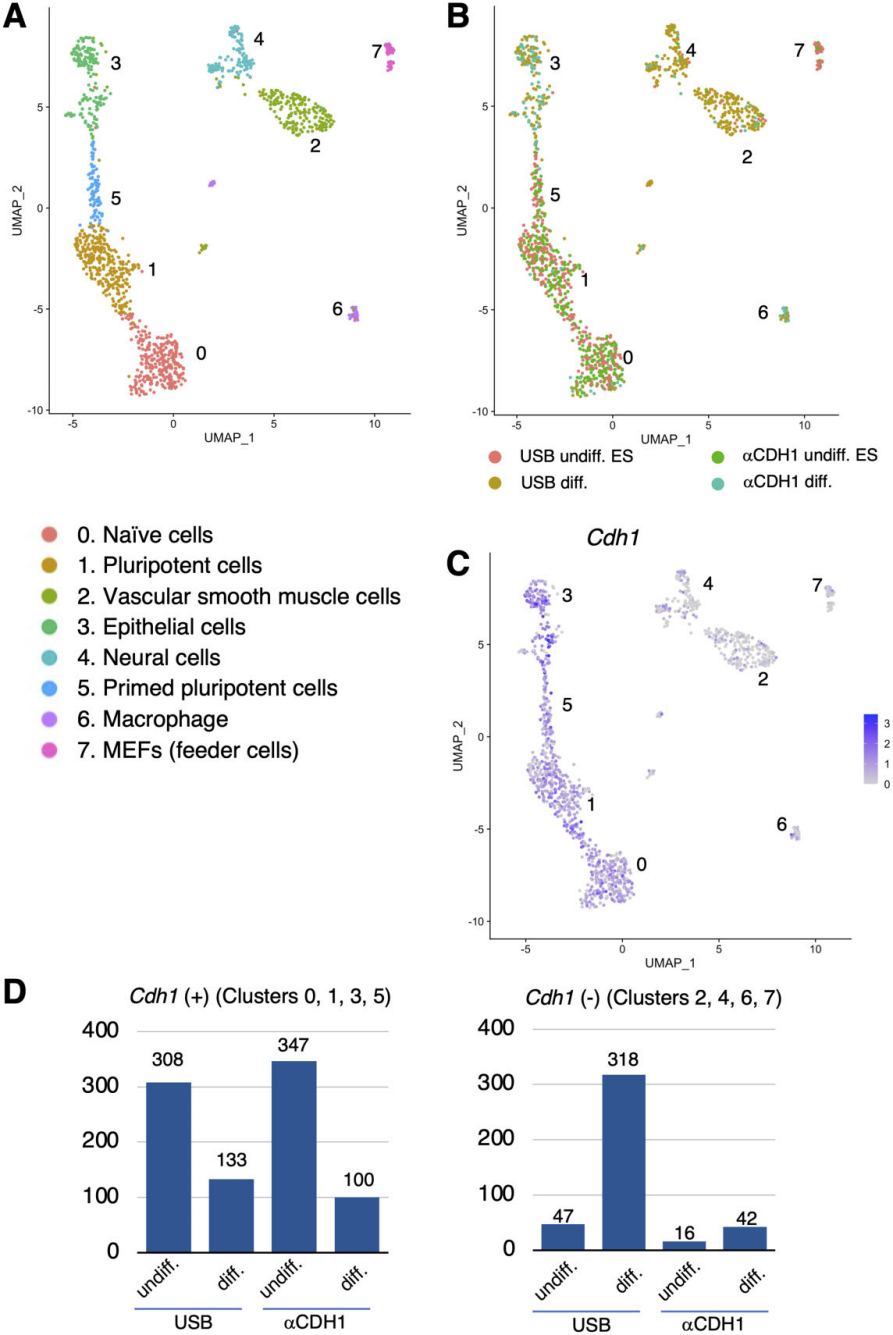
scRNA-seq analysis of ES cells and their differentiated derivatives using the USB and Ab multiplexing methods. (A) UMAP analysis classified cells into eight clusters: 0, naïve cells; 1, pluripotent cells; 2, vascular smooth muscle cells; 3, epithelial cells; 4, neural cells; 5, primed pluripotent cells; 6, macrophage; 7, MEFs (feeder cells). (B) Distribution of four cell samples on the UMAP. The colour code for each sample is shown at the bottom of the figure. (C) Distribution of *Cdh1*-positive cells on the UMAP. (D) Comparisons of number of cells detected by the USB or the Ab method in *Cdh1*-positive (left) and *Cdh1*-negative (right) clusters.

After eliminating dead cells and doublets (see Section 2), informatics analysis was performed on the remaining 2,004 cells (Table 1, Supplementary Table S2). Data obtained from the undifferentiated and differentiated states of the R1 ES cell line labelled with either the USB or the Ab methods are illustrated (Fig. 2). Cells were classified into 8 clusters using the UMAP method. Based on the genes expressed, Clusters 0, 1 and 5 corresponded to pluripotent cells, naïve cells and primed pluripotent cells, respectively and were considered as undifferentiated cells (Fig. 2A, Table 2). It is noteworthy that cells in these clusters labelled by the two different methods were classified into the same cluster with similar distribution patterns (Supplementary Fig. S3A and B), i.e. the expression profile of the cells labelled by the USB method was highly similar to that of the cells labelled by the conventional Ab method. Furthermore, differential gene expression analyses were performed to assess changes in transcriptome between the USB-treated and the Ab-treated undifferentiated ES cells. Both non-parametric Wilcoxon rank sum test and MAST^15^ of Seurat^13^^,^^14^ were used to examine differences between the USB-treated and Ab-treated cells within *Cdh1*-positive clusters, i.e. Clusters 0, 1, 3, 5. Both methods did not reveal statistically significant differentially expressed genes between the two samples (see Supplementary Table S3). Therefore, it is evident that the USB method does not cause any discernible differences in transcriptome compared with the one obtained by the Ab method.

**Table 1:** Number of cells detected by multiplex scRNA-seq in undifferentiated and differentiated R1 ES cells

Tag ID	Cells	Labelling methods	Total cell no.	*Cdh1*(+) cell no. (Clusters 0, 1, 3 and 5)	*Cdh1*(−) cell no. (Clusters 2, 4, 6 and 7)
A951	R1 undiff. ESC	NHS-Biotin labelling	355	308 (86.8%)	47 (13.2%)
A952	R1 diff. ESC	NHS-Biotin labelling	451	133 (29.5%)	318 (70.5%)
A953	R1 undiff. ESC	Biotin-anti-E-Cadherin labelling	363	347 (95.6%)	16 (4.4%)
A954	R1 diff. ESC	Biotin-anti-E-Cadherin labelling	142	100 (70.4%)	42 (29.6%)
			Total = 1,311		

**Table 2:** List of UMAP clusters from the analysis of undifferentiated and differentiated R1 cells and Top 20 of identified marker genes belong to each cluster

Cluster ID	Top 20 of identified marker genes	Cell types	*Cdh1* + or −
0	*Dppa5a, Zfp42, Fbxo15, Tdh, Zfp600, Dnmt3l, Mybl2, Chchd10, Gm47654, Klf2, Ppcdc, Zfp990, Rps4l, Mkrn1, Dhx16, Hspb1, Sgk1, Platr3, Mycn, Rhox5*	Naïve cells	+
1	*Pim2, Dnmt3b, Gng3, Car2, Gm19792, Pou3f1, Npm1, Olfr1388, 2810429I04Rik, Cd59b, Pou5f1, Wnt8a, Snrpn, Tdgf1, Psat1, Dut, L1td1, Ldha, Olfr1459, Trh*	Pluripotent cells	+
2	*Cdh11, Igf2, Col3a1, Gm49394, Igfbp4, Fstl1, Hmga2, Peg3, Col1a1, Dlk1, Mest, Col1a2, Itm2a, H19, Ptn, Acta2, Postn, Plagl1, Tagln, Actg2*	Vascular smooth muscle cells	−
3	*Krt19, Krt8, Cldn6, Krt18, Perp, Dsc2, Anxa2, F3, Sfn, Ccnd2, Anxa1, Krt15, Krt6a, Fam107b, Mt1, Rbp4, Clu, Podxl, Spink1, Afp*	Epithelial cells	+
4	*Nr2f1, Map2, Sox11, Ckb, Map1b, Ttc3, Fabp7, Vezf1, Tubb2b, Sox21, Zic1, Shh, H1f0, 6330403K07Rik, Nnat, Cdk5r1, Slit2, Jam2, Spon1, Tubb3*	Neural cells	−
5	*Gsc, Cer1, Fgf5, Lhx1, Cyp26a1, T, Flt1, Lefty1, Eomes, Trh, Car4, Foxj1, Igfbp3, Upp1, Ndrg1, Emb, Lefty2, Apela, Mns1, Amot*	Primed pluripotent stem cells	+
6	*Evi2a, Fcer1g, C3ar1, Tyrobp, C1qc, C1qb, Nfam1, Mpeg1, Laptm5, C1qa, Ptprc, Lyz2, Lgmn, Ctsb, Lpl, Plp1, Moxd1, Adcy7, Ctsd, Cxcl16*	Macrophage	−
7	*Crabp1, Ly6a, Ccl7, S100a4, Ccl2, Lrrc15, Fbln2, Bgn, Thy1, Dcn, Tnc, Timp1, S100a6, Gsto1, Lgals1, Emp1, AC109138.2, Spp1, Mmp3, AC160336.1*	MEFs (feeder cells)	−

Clusters other than 0, 1 and 5 were identified as differentiated cells, such as vascular smooth muscle cells (Cluster 2), epithelial cells (Cluster 3), neural cells (Cluster 4) and macrophage (Cluster 6). MEF feeder cells that could not be completely removed were detected as Cluster 7. A breakdown of the cell samples belonging to each cluster revealed that cells labelled by the USB method were present in almost all clusters (Fig. 2A and B, Supplementary Fig. S3A–F), indicating that this method can indeed capture various types of differentiated cells.

In contrast, the number of cells labelled by the Ab method was clearly reduced in differentiated cell clusters 2 and 4 (Fig. 2B, Supplementary Fig. S3C, D and F), probably due to down-regulation of *Cdh1* gene expression in these cells (Fig. 2A and C). The differentiated cells labelled with the USB method were more uniformly distributed in these clusters. Similar numbers of cells in the *Cdh1*-positive clusters (Clusters 0, 1, 3 and 5) were labelled by the USB and Ab methods. However, in the *Cdh1*-negative clusters (Clusters 2, 4, 6, 7), the number of cells labelled with the Ab method was clearly lower than with the USB method (Fig. 2D, Supplementary Table S4), indicating the limitation of the Ab method for detecting differentiated cells in this experiment.

This trend was also observed for the EB3 line, indicating that the USB method can be applied to different ES cell lines (Supplementary Fig. S4A–I, Supplementary Tables S2, S4 and S5).

## 4. Discussion

We have developed a new, simple and reliable cell labelling method for multiplexing in scRNA-seq analysis and compared its performance with that of the Ab method widely used multiplexing method. The Ab method^6^ targets specific proteins that are ubiquitously expressed on the surface of cells to be tested, and thus are difficult to use when such proteins are not expressed ubiquitously in the samples. The USB method, however, does not require such specific proteins, but rather uses universal labelling that can target any proteins, thus overcoming the shortcomings of the existing techniques. The USB method can indeed capture a variety of differentiated cells, whereas the Ab method cannot capture some of these differentiated cells. Furthermore, differential gene expression testing revealed that the USB method does not cause any discernible differences in transcriptome compared with the one obtained by the Ab method.

Multiplexing reagents currently available use antibodies to some cell surface proteins such as CD298 and/or MHC class I antigens or CD45, because these antigens are thought to be expressed ubiquitously in adult tissues or in the immune system. However, embryonic cells do not express these proteins, and thus the current cell hashing methods are not applicable. The USB method, however, is compatible with all cell surface proteins and can be used for multiplexing regardless of the cell types involved (as long as the cells have surface proteins). In fact, we successfully applied the USB method to multiplexing of chondrocytes from mouse, monkey and human iPS,^17^ as well as rat lung cells, which were not able to be studied with the Ab method using CD45 or MHC antibodies (Supplementary Fig. S5A and B, data not shown). It should be noted that the USB method can be applied to species other than mouse or human. For other species, information about the cell surface proteins or antibodies against them may not be readily available, and therefore it would be difficult to apply conventional Ab methods. However, the USB method is proven to be applicable for monkey and rat, and in theory, can be used for cells of any species.

Other labelling technologies for multiplexing have been developed, including the transient barcoding method,^18^ in which barcode DNA is transfected into cells, and the CellTag Indexing method, which uses lentivirus for barcode DNA transduction.^19^ However, these methods are difficult to apply to cells other than cultured cells, and hence their versatility is limited. In contrast, the USB method is extremely versatile and applicable to any cell type. In addition, the ClickTag multiplexing method,^20^ which combines click chemistry and NHS-ester chemistry, is a technology that attaches barcode DNA tags to cellular proteins in a manner similar to the USB method. However, the labelling efficiency of live cells is poor and the method can only be applied to methanol-fixed cells. The CellPlex method (i.e. 3′ CellPlex Kit, 10× Genomics) uses reagents to introduce a DNA tag to lipids of the cell membrane for universal cell labelling,^21^ but it is difficult to apply to methanol-fixed cells. We are currently developing a modified USB protocol for methanol-fixed samples and have obtained results demonstrating that the USB method can be applied to fixed cells (unpublished), allowing even more flexible sample multiplexing.

*Cdh1*-negative cells are supposedly not labelled with anti-CDH1 antibody and should be eliminated during the analysis. However, some cells were actually classified as *Cdh1*-negative (see Fig. 2), even though the number of *Cdh1*-negative cells was clearly reduced in the Ab treated cells (Table 1). A possible reason for this was non-specific binding of antibodies or streptavidin to the cells. In fact, we observed that hashtag DNA was amplified in both streptavidin-only and isotype control antibody-treated samples (Supplementary Fig. S6). Since the hashtag DNA was not amplified at all in the unlabelled samples, the amplification observed in the streptavidin-only and isotype control antibody-treated samples (Supplementary Fig. S6D and E, arrow) was likely due to non-specific binding of the streptavidin and/or isotype control antibody to the cells. The amplification by scRNA-seq procedure may be more sensitive than flow cytometry, and therefore, cells that do not express CDH1 (or express very low amounts) may have been classified as positives. However, for cell labelling in scRNA-seq multiplexing, it is very important that all cells in the sample are labelled uniformly, so a small amount of non-specific binding should not affect the multiplexing results.

As described in Section 2, all the components used in the USB method are commercially available and are relatively inexpensive. The stock solution of S-NHS-biotin is stable at –80°C for more than 1 year and a vial containing 50 mg of S-NHS-biotin is sufficient for labelling of ∼10,000 samples. Ten micrograms of the hashtag-streptavidin can be used for >150 multiplexing experiments. Since 10 types of hashtag-streptavidin are commercially available, it is possible to perform 10-plex analysis at one time. As custom hashtag oligo DNA can be produced, it will be possible to conduct more than 10-plex experiments. In summary, the USB method is a cost-effective method that makes single-cell multiplexing analysis more affordable and approachable, and we propose that the USB method is the universal labelling method that can be applied to any type of cells, live or fixed and will greatly facilitate any type of multiplexing scRNA-seq analysis.

## Supplementary Material

dsac017_Supplementary_Data
