# Supplementary material for: Universal Surface Biotinylation: a simple, versatile and cost-effective sample multiplexing method for single-cell RNA-seq analysis
Source: DNA Res. 2022 Jun 2;29(3):dsac017. doi: 10.1093/dnares/dsac017 (PMC9202638; doi:10.1093/dnares/dsac017)
Supplement: dsac017_Supplementary_Data [file dsac017_supplementary_data.zip › FigS1-5_0501.pptx]

## Slide 1
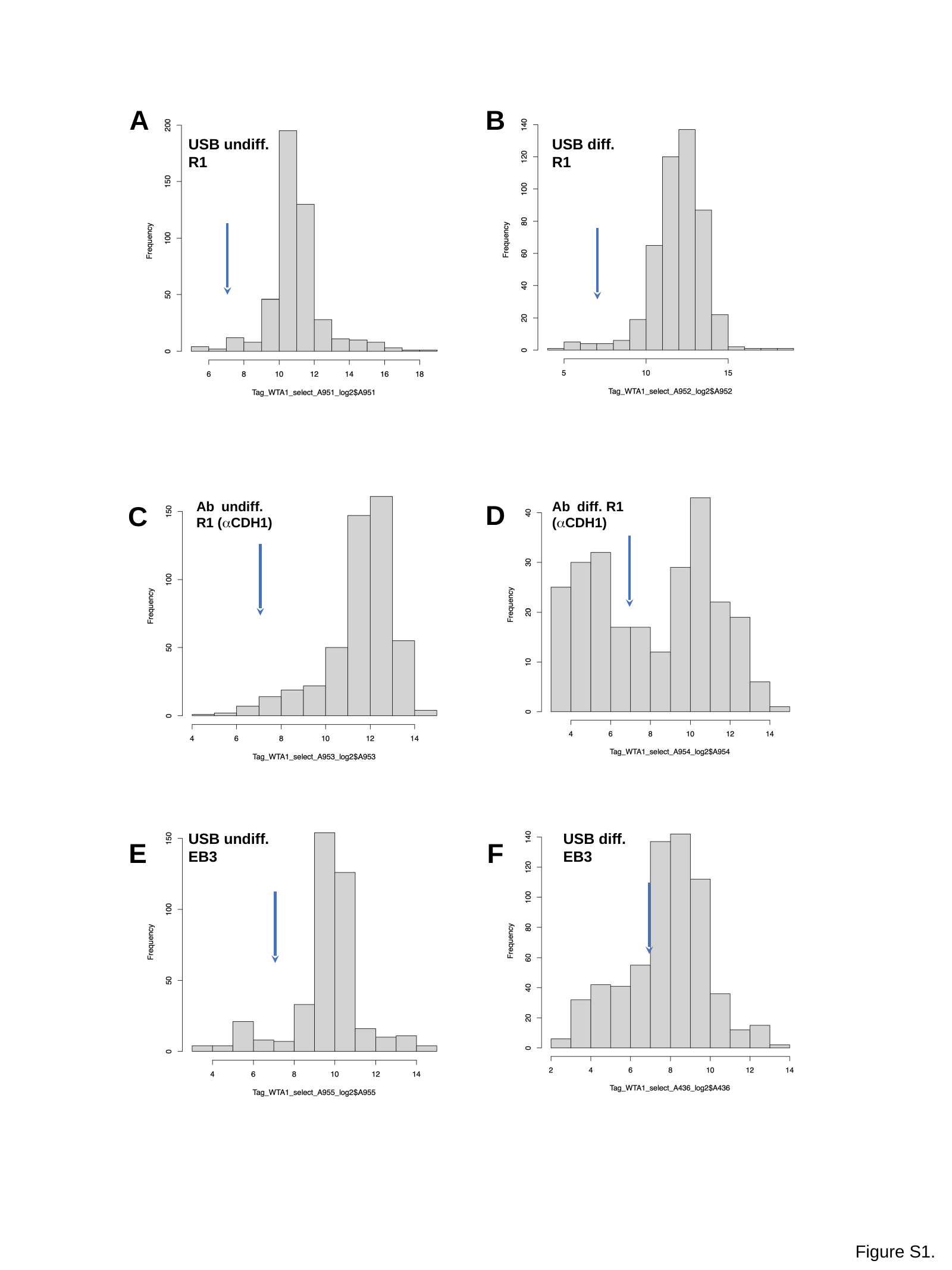

A
B
USB undiff. R1
USB diff. R1
D
C
Ab undiff. R1 (aCDH1)
Ab diff. R1
(aCDH1)
USB undiff. EB3
USB diff. EB3
E
F
Figure S1.

## Slide 2
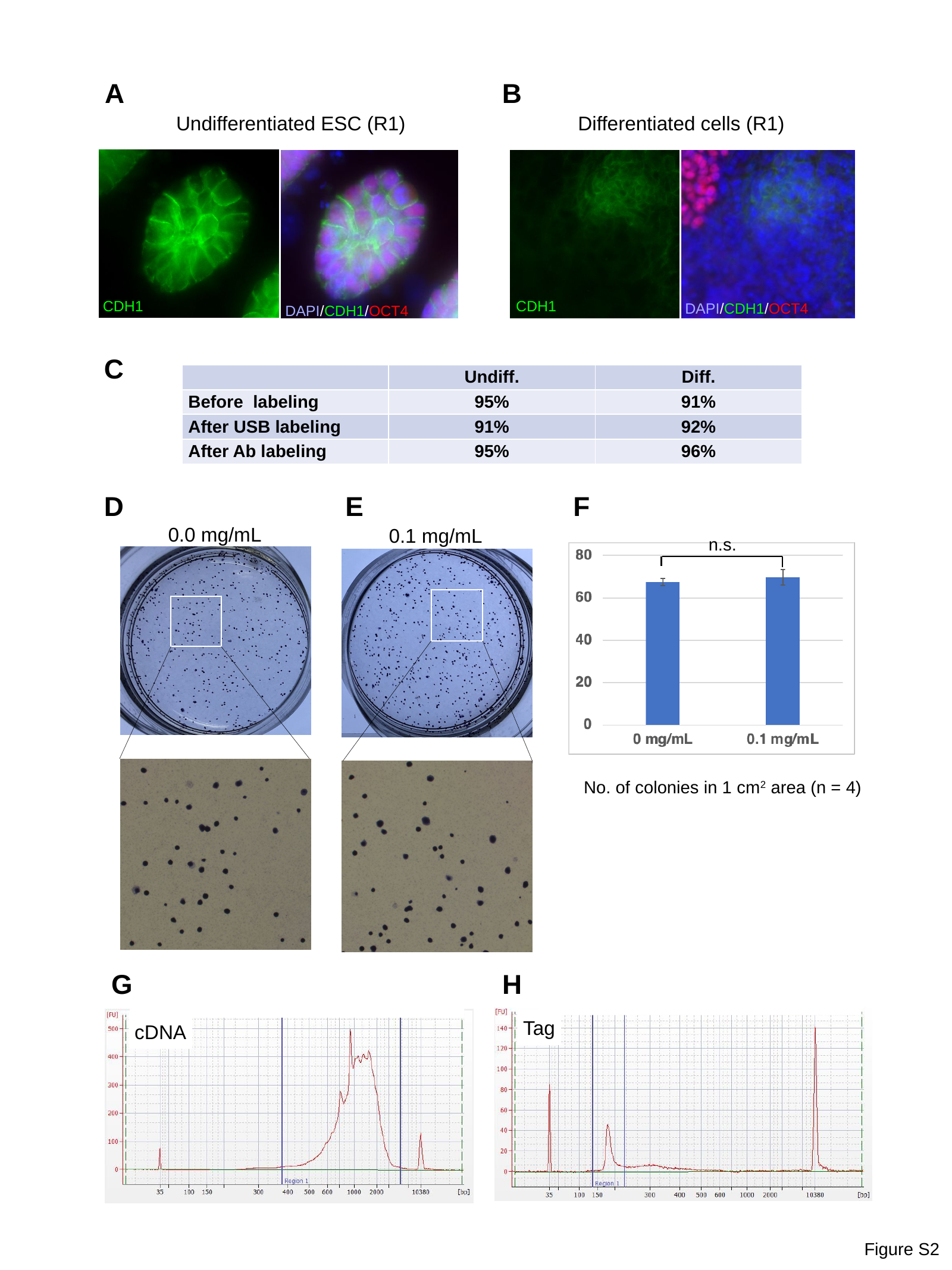

A
B
Undifferentiated ESC (R1)
Differentiated cells (R1)
CDH1
CDH1
DAPI/CDH1/OCT4
DAPI/CDH1/OCT4
C
| | Undiff. | Diff. |
| --- | --- | --- |
| Before labeling | 95% | 91% |
| After USB labeling | 91% | 92% |
| After Ab labeling | 95% | 96% |
D
E
F
0.0 mg/mL
0.1 mg/mL
n.s.
No. of colonies in 1 cm2 area (n = 4)
G
H
Tag
cDNA
Figure S2

## Slide 3
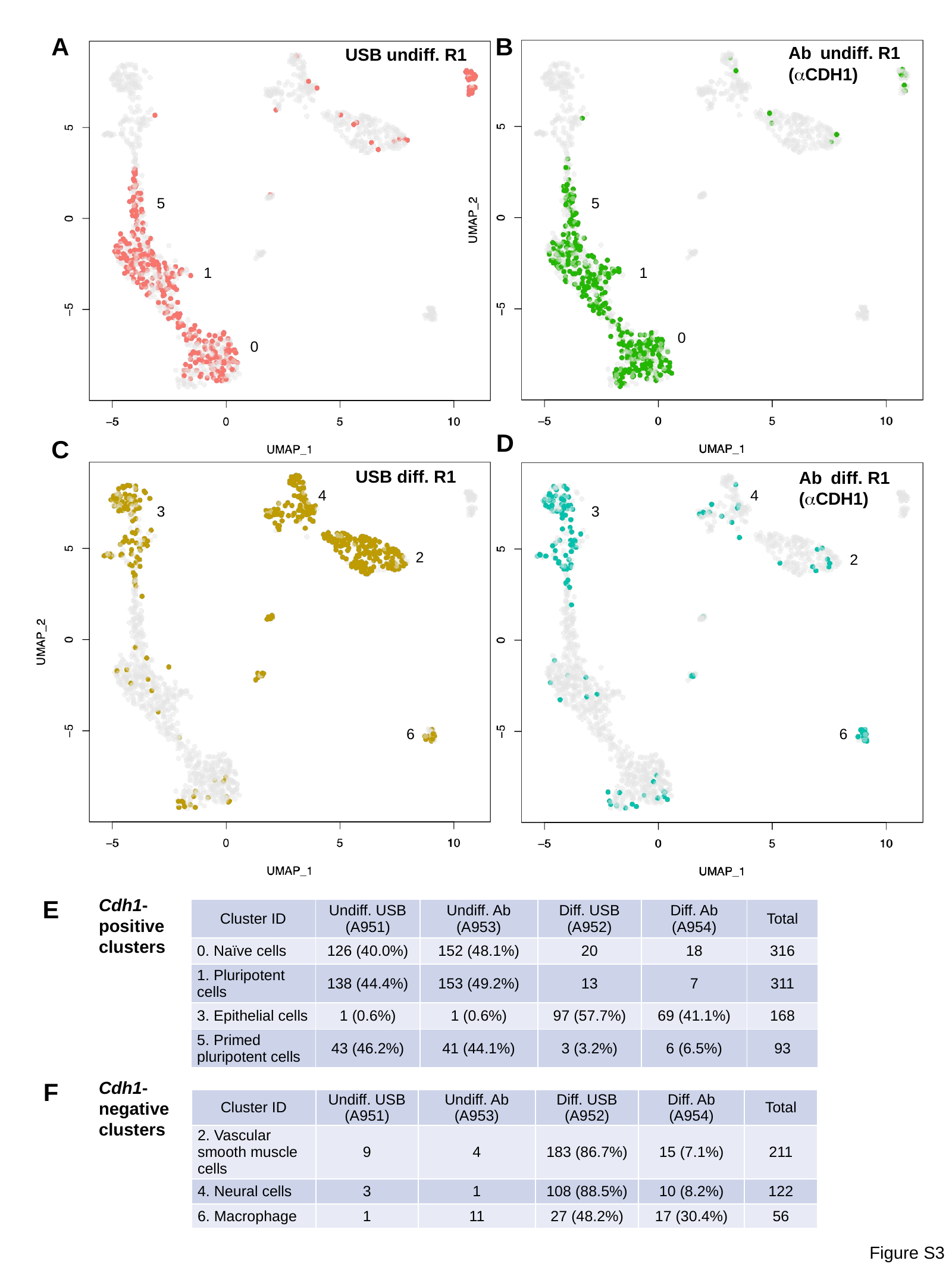

A
B
Ab undiff. R1
(aCDH1)
USB undiff. R1
5
5
1
1
0
0
D
C
USB diff. R1
Ab diff. R1
(aCDH1)
4
4
3
3
2
2
6
6
E
Cdh1-positive clusters
| Cluster ID | Undiff. USB (A951) | Undiff. Ab (A953) | Diff. USB (A952) | Diff. Ab (A954) | Total |
| --- | --- | --- | --- | --- | --- |
| 0. Naïve cells | 126 (40.0%) | 152 (48.1%) | 20 | 18 | 316 |
| 1. Pluripotent cells | 138 (44.4%) | 153 (49.2%) | 13 | 7 | 311 |
| 3. Epithelial cells | 1 (0.6%) | 1 (0.6%) | 97 (57.7%) | 69 (41.1%) | 168 |
| 5. Primed pluripotent cells | 43 (46.2%) | 41 (44.1%) | 3 (3.2%) | 6 (6.5%) | 93 |
F
Cdh1-negative clusters
| Cluster ID | Undiff. USB (A951) | Undiff. Ab (A953) | Diff. USB (A952) | Diff. Ab (A954) | Total |
| --- | --- | --- | --- | --- | --- |
| 2. Vascular smooth muscle cells | 9 | 4 | 183 (86.7%) | 15 (7.1%) | 211 |
| 4. Neural cells | 3 | 1 | 108 (88.5%) | 10 (8.2%) | 122 |
| 6. Macrophage | 1 | 11 | 27 (48.2%) | 17 (30.4%) | 56 |
Figure S3

## Slide 4
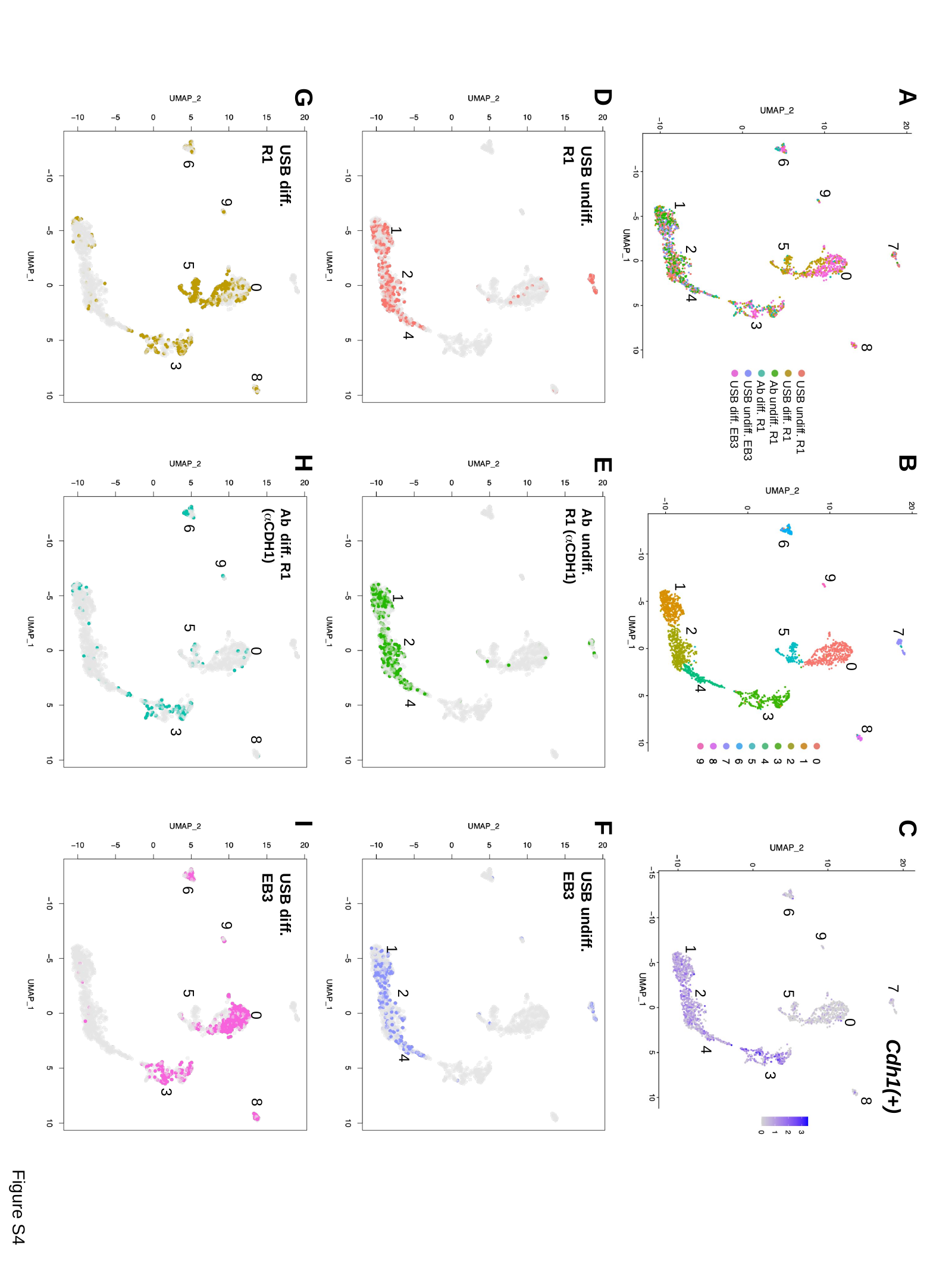

D
A
G
6
6
USB diff. R1
USB undiff. R1
9
9
1
1
7
5
2
5
2
0
0
4
3
4
8
3
USB undiff. R1
USB diff. R1
Ab undiff. R1
Ab diff. R1
USB undiff. EB3
USB diff. EB3
8
H
B
E
Ab diff. R1
(aCDH1)
Ab undiff. R1 (aCDH1)
6
6
9
9
1
1
5
2
5
7
2
0
0
4
4
3
8
3
8
I
F
C
6
USB diff. EB3
USB undiff. EB3
6
9
9
1
1
7
5
2
2
5
0
0
4
4
3
3
8
8
Cdh1(+)
Figure S4

## Slide 5
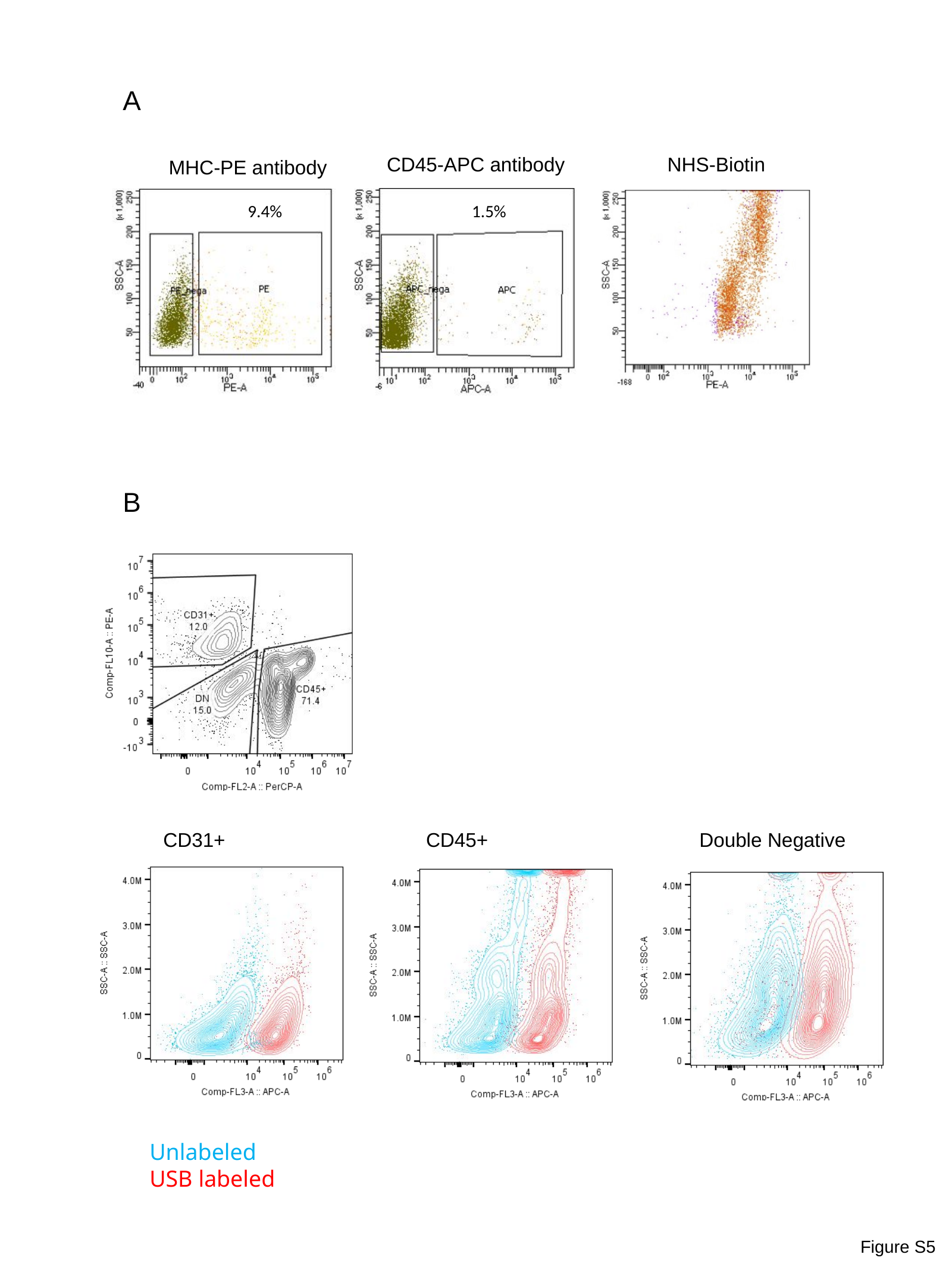

A
CD45-APC antibody
NHS-Biotin
MHC-PE antibody
9.4%
1.5%
B
CD31+
CD45+
Double Negative
Unlabeled
USB labeled
Figure S5

## Slide 6
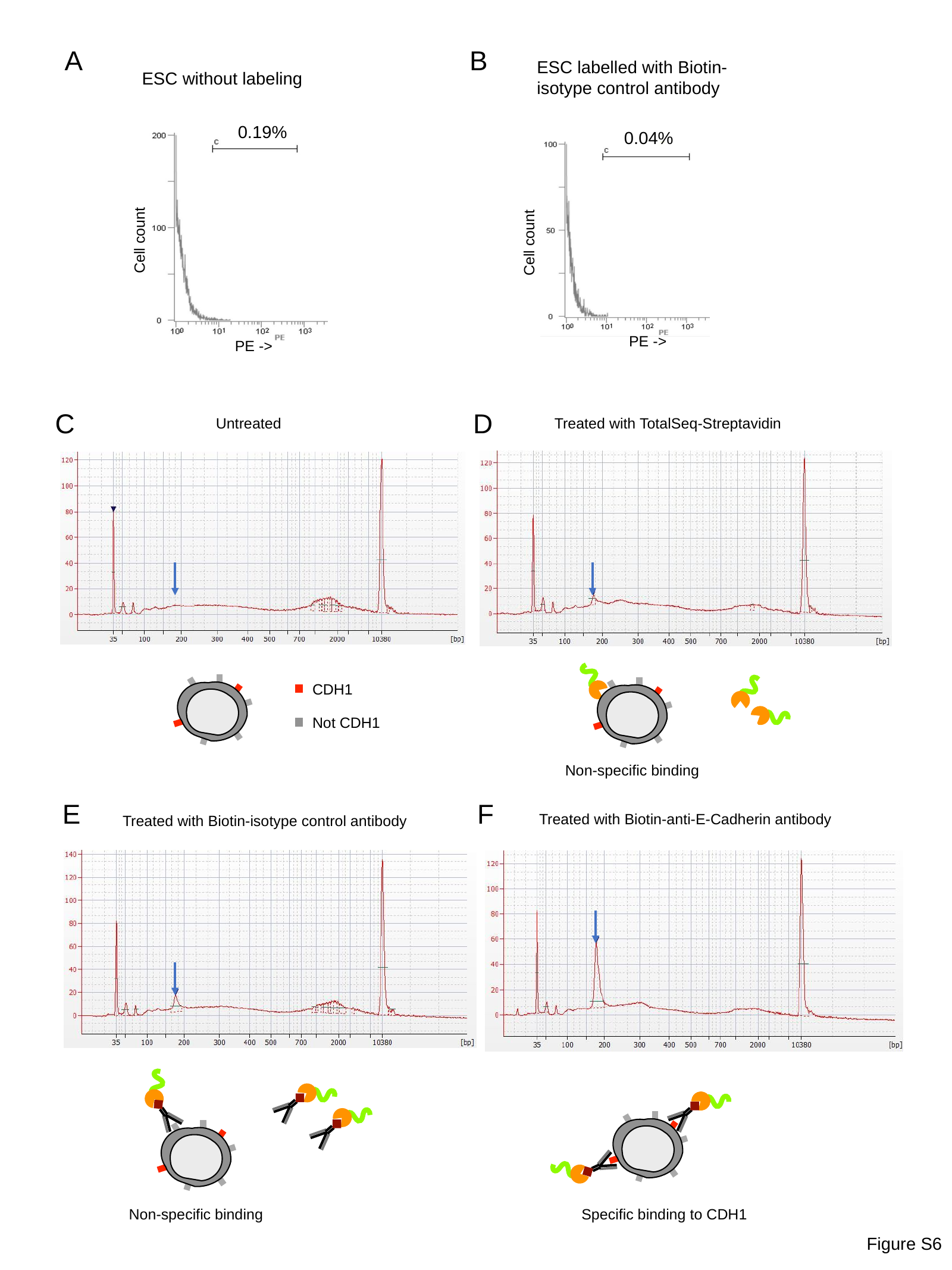

A
B
ESC labelled with Biotin-
isotype control antibody
ESC without labeling
0.19%
0.04%
Cell count
Cell count
PE ->
PE ->
C
D
Untreated
Treated with TotalSeq-Streptavidin
CDH1
Not CDH1
Non-specific binding
E
F
Treated with Biotin-anti-E-Cadherin antibody
Treated with Biotin-isotype control antibody
Non-specific binding
Specific binding to CDH1
Figure S6
