# Supplementary material for: Universal Surface Biotinylation: a simple, versatile and cost-effective sample multiplexing method for single-cell RNA-seq analysis
Source: DNA Res. 2022 Jun 2;29(3):dsac017. doi: 10.1093/dnares/dsac017 (PMC9202638; doi:10.1093/dnares/dsac017)
Supplement: dsac017_Supplementary_Data [file dsac017_supplementary_data.zip › Supplemental Data (legends).docx]

**Supplemental Data**

**Tables**

Table S1.

Table S1a. List of hashtag-conjugated Streptavidin or Antibody used in this study and barcode sequences within the hashtag DNA,

Table S1b. List of primers used for TAS-seq amplification.

Table S2.

Number of cells detected by multiplex scRNA-seq in undifferentiated and differentiated R1 and EB3 ES cells.

Table S3.

Table S3a. Differentially expressed genes between USB-treated cells and Ab-treated cells analyzed by Seurat default method.

Table S3b. Differentially expressed genes between USB-treated cells and Ab-treated cells analyzed by MAST.

Table S4.

Table S4a. Number of cells belong to the UMAP clusters from multiplexed samples (R1 cell line).

Table S4b. Number of cells belong to the UMAP clusters from multiplexed samples (R1 and EB3 cell lines).

Table S5.

List of UMAP clusters from the analysis of undifferentiated and differentiated R1 and EB3 cells and Top 20 of identified marker genes belong to each cluster.

**Figures**

Figure S1.

Histogram of multiplex hashtag counts in each sample.

(A) Distribution of hashtag counts in the USB-labeled undifferentiated R1 ES cells. (B) Distribution of hashtag counts in the USB-labeled differentiated R1 cells. (C) Distribution of hashtag counts in the Ab-labeled undifferentiated R1 ES cells. (D) Distribution of hashtag counts in the Ab-labeled differentiated R1 cells. (E) Distribution of hashtag counts in the USB-labeled undifferentiated EB3 ES cells. (F) Distribution of hashtag counts in the USB-labeled differentiated EB3 cells.

A single peak is detected in these histograms except for (D). In case of (D), i.e., the Ab-treated differentiated R1 cells, an additional peak with smaller number of hashtag counts is detected, and we suspect that this peak might correspond to the cells labeled non-specifically by the anti-CDH1 antibody or to cells with scarce target molecules, and that these cells cannot be used reliably for the cell multiplexing. We set the threshold value (blue arrow) using mclust package in R [1] to separate two peaks, and the cells with the counts above the threshold were used for the subsequent analysis. For USB-treated differentiated EB3, the peak is shifted toward lower tag counts, may be due to differences in affinity to biotin between streptavidin and anti-biotin antibody used for this analysis.

Figure S2.

Immunostaining of CDH1 for (A) undifferentiated ES cells and (B) differentiated cells. CDH1 signals were observed on the cell surface of all undifferentiated ES cells, while the signals were detected in only a small number of differentiated cells. Green, CDH1; red, OCT4; blue, DAPI (nucleus). (C) Cell viability before and after USB or Ab treatment. Cell viability was measured by Trypan Blue assay. (D,E) S-NHS-biotin treatment does not affect the viability of undifferentiated ES cells. Cells treated with (E) S-NHS-biotin and (D) untreated control. (F) Comparison of colony numbers between S-NHS-Biotin treated cells (E) with untreated control (D). The numbers represent colony number per 1 cm^2^ area. Student-t test reveals no significant differences between the two samples (p=0.114). n.s. ; not significant.

(G, H) Quality check of (G) whole-transcriptome cDNA, and (H) hashtag DNA amplified by using the TAS-seq protocol. According to the electrophoretogram, the amount and size distribution of cDNA and hashtag DNA were satisfactory for the subsequent analysis.

Figure S3.

(A) Distribution of USB-labeled undifferentiated R1 ES cells on the UMAP.

(B) Distribution of Ab-labeled undifferentiated R1 ES cells on the UMAP.

Cluster number of *Cdh1*-positive clusters, i.e., 0, 1, 5, in undifferentiated ES cells are indicated in (A) and (B).

(C) Distribution of USB-labeled differentiated R1 cells on the UMAP.

(D) Distribution of Ab-labeled differentiated R1 cells on the UMAP. Cluster number of *Cdh1*-negative clusters, i.e., 2, 4, 6 in differentiated R1 cells are indicated in (C) and (D).

(E) Number of cells belong to *Cdh1*-positive clusters 0,1,3,5 in four samples, i.e., Undifferentiated R1 ES cells USB treated, Undifferentiated R1 ES Ab treated, Differentiated R1 USB treated, and Differentiated R1 Ab treated, are tabulated.

(F) Number of cells belong to *Cdh1*-negative clusters 2, 4, 6 in the USB treated Differentiated cells and Ab treated Differentiated cells are tabulated.

Data of MEF (Cluster 7: contaminated feeder cells) were omitted.

Figure S4.

Multiplex scRNA-seq analysis of six samples.

The six samples include four samples from R1 ES cells, undifferentiated and differentiated cells labeled by either the USB or the Ab method, and two samples from the EB3 ES cell line, undifferentiated and differentiated, labeled by the USB method. Cell clustering was done by UMAP and the distribution of each sample is shown in (A).

(B) Ten clusters were identified as follows. 0, vascular smooth muscle; 1, naive ES cells; 2, pluripotent cells; 3, epithelial cells; 4, primed pluripotent stem cells; 5, neural cells; 6, macrophage; 7, MEFs (feeder cells); 8, glial cells; 9, vascular endothelial cells.

(C) Overlay of *Cdh1* expression level onto the UMAP.

(D) Distribution of USB-labeled undifferentiated R1 ES cells on the UMAP.

(E) Distribution of Ab-labeled undifferentiated R1 ES cells on the UMAP.

(F) Distribution of USB-labeled undifferentiated EB3 ES cells on the UMAP.

Cluster number of *Cdh1*-positive clusters, i.e., 1, 2, 4, in undifferentiated R1 and EB3 cells are indicated in (D)-(F).

(G) Distribution of USB-labeled differentiated R1 cells on the UMAP.

(H) Distribution of Ab-labeled differentiated R1 cells on the UMAP.

(I) Distribution of USB-labeled differentiated EB3 cells on the UMAP. Cluster number of *Cdh1*-negative clusters, i.e., 0, 5, 6, 8, 9, in differentiated R1 and EB3 cells are indicated in (G)-(I).

Figure S5.

Examples of cell samples for which the conventional multiplexing method is not applicable.

(A) Flow cytometry analysis of chondrocytes from mouse growth plate labeled with MHC Class I antibody (left), CD45 antibody (middle), and USB method (right). It was confirmed that mouse growth plate chondrocytes, which were poorly stained by MHC and CD45 antibodies, were labeled efficiently by the USB method.

(B) Flow cytometry analysis of rat lung cells. Dissociated cells were fractionated into three populations, i.e., CD31-positive, CD45-positive and double negatives. All the fractionated cells were found to be labeled by the USB method. Rat is a species for which the sample multiplexing kit based on the Ab method is not commercially available.

Figure S6.

Nonspecific binding of antibodies and hashtag- streptavidin (TotalSeq-streptavidin) in the Ab-based analysis.

(A, B) Results of flow cytometry analysis. (A) Unlabeled undifferentiated ES cells; (B) ES cells treated with biotin–isotype control antibody and TotalSeq-streptavidin (PE-conjugated).

(C–F) Nonspecific binding of antibody/TotalSeq-streptavidin detected by the TAS-seq method.

(C) Cells not treated with either antibody or TotalSeq-streptavidin.

(D) Cells treated with TotalSeq-streptavidin only.

(E) Cells treated with isotype control antibody followed by TotalSeq-streptavidin.

(F) Cells treated with biotin-conjugated anti-CDH1 antibody, and then TotalSeq-streptavidin.

A schematic representation of the relationship of the sample reagents is shown at the bottom of each panel (see also legend of Figure 1A).

From each sample, mRNA was collected, and cDNA was synthesized; hashtag DNA was amplified as described in the Materials and Methods section. Samples were then analyzed using the Agilent Bioanalyzer. In the negative control sample (C), no amplification was detected on the electrophoretogram, while a clear peak (arrow) was observed in the positive control (F). In other samples (D and E), although smaller than that in (F), peaks were observed (arrow), indicating the presence of nonspecific binding of TotalSeq-streptavidin and/or isotype control antibody.

**Supplemental Reference**

[1] Scrucca L., Fop M., Murphy T. B., and Raftery A. E. 2016, mclust 5:

clustering, classification and density estimation using Gaussian

  finite mixture models, The R Journal, 8, 289-317.
